# Comparative structural analysis of retroviral fusion proteins identifies regions that modulate membrane fusion: a potential retroviral achilles heal?

**DOI:** 10.1186/1742-4690-8-S1-A163

**Published:** 2011-06-06

**Authors:** Daniel Lamb, Alexander W Schüttelkopf, Daan M F van Aalten, David W Brighty

**Affiliations:** 1 The Biomedical Research Institute, College of Medicine, Ninewells Hospital, University, Dundee, Scotland, DD1 9SY, UK; 2 Division of Molecular Microbiology, College of Life Sciences, University of Dundee, Dundee, DD1 5EH2, UK

## 

Refolding of viral class-1 membrane fusion proteins from a native state to a trimer-of-hairpins structure promotes entry of viruses into cells. Here we present the structure of the bovine leukaemia virus transmembrane glycoprotein (TM) and identify a group of asparagine residues at the membrane-distal end of the trimer of hairpins that is strikingly conserved among divergent viruses. These asparagines are not essential for surface display of pre-fusogenic envelope. Instead, substitution of these residues dramatically disrupts membrane fusion. Our data indicate that through electrostatic interactions with of a chloride ion the asparagine residues promote assembly and profoundly stabilize the fusion-active structures that are required for viral envelope-mediated membrane fusion. Moreover, the BLV TM structure also reveals a charge-surrounded hydrophobic pocket on the central coiled coil and interactions with basic residues that cluster around this pocket are critical to membrane fusion and form a target for peptide inhibitors of envelope function. Charge-surrounded pockets and electrostatic interactions with small ions are common leitmotifs among class-1 fusion proteins. We will discuss the impact of these observations in light of current models of membrane fusion and as potential targets for therapeutic inhibition of viral entry.

